# Empowered listening: Self-selected music’s role in uplifting emotions during stress

**DOI:** 10.3389/fpsyg.2026.1757012

**Published:** 2026-06-16

**Authors:** Caitlin Fountain, Jeffrey J. Klibert, Laura A. Stambaugh, Kaitlin Bountress, Saranga Bansal, Lawrence Locker

**Affiliations:** 1 Department of Psychology, Virginia Commonwealth University, Richmond, VA, United States; 2 Department of Psychology, Georgia Southern University, Statesboro, GA, United States; 3 Department of Music, Rhode Island College, Providence, RI, United States; 4 Virginia Institute for Psychiatric and Behavioral Genetics, Virginia Commonwealth University, Richmond, VA, United States

**Keywords:** emotion regulation, music psychology, positive psychology, self-selected music, stress

## Abstract

**Introduction:**

Music listening is increasingly recognized not only as a form of entertainment but a complementary intervention for well-being. Its ability to promote relaxation and reduce arousal, particularly following stress, highlights its therapeutic potential. However, the differential effects of various types of music on emotional regulation in response to stress remain underexplored, limiting the development of musically derived interventions. The present study examined the effects of self-selected music listening (high valence/high tempo [empowering], high valence/low tempo [calming]) on emotion regulation processes (upregulation of positive emotions, downregulation of negative emotions) following a social rejection stress task.

**Methods:**

A multi-phase research design was employed. In Phase 1 (musical preferences screener), 795 undergraduate students in the United States completed the Short Test of Music Preferences (STOMP) and Music Rating Form. These data were used to create individualized playlists consisting of self-selected empowering (high valence/high tempo) and calming (high valence/low tempo) songs. In the experimental phase (Phase 2), 61 participants from Phase 1 were randomly assigned to one of three conditions (empowering music, calming music, control group) following a stress induction task. Measures of positive and negative emotions (PANAS) were administered at three time points during the experiment.

**Results:**

The stress induction task was effective in increasing participants’ stress levels. Results from the primary analyses revealed a significant interaction between time and music listening intervention group for positive emotions. Specifically, participants in the empowering music listening group reported significantly higher positive emotion scores at post-intervention (Time 3) compared to those in the control group. This effect was not observed for participants in the calming music group. In contrast, the time by intervention condition interaction was not significant for negative emotions.

**Discussion:**

These findings suggest listening to empowering music may serve as an effective and accessible practice for uplifting positive emotions in response to stressors. Notably, this study contributes to the field by demonstrating how specific features of musical interventions (self-selected playlists, distinct musical qualities) can optimize positive emotional outcomes, especially in the context of stress. Overall, these results support the integration of music-based interventions into holistic, strength-based therapeutic practices aimed at improving emotional health.

## Introduction

As rates of psychological distress among transitional youth (18- to 25-year-olds) continue to rise (Healthy Minds Network, 2026), the emerging field of music emotion regulation (MER) offers an evidence-based, accessible, and person-centered approach to enhancing and maintaining well-being (Chi and Tan, 2025). Central to MER is emotion regulation (ER), defined as the purposeful process of influencing the intensity, duration, and scope of emotional experiences (Gross, 1998, 2015). In general, ER aims to help individuals flexibly augment their experience of an emotional state to a more manageable, adaptable, or psychologically beneficial state (Gross, 2015), thereby supporting overall well-being (Saccaro et al., 2024). The mechanisms underlying ER are complex and vary depending on individual need and contextual factors. For instance, obtaining emotional balance may involve increasing positive emotions (upregulation), decreasing negative emotions (downregulation), modulating emotional intensity, extending emotional duration, or altering the quality of emotions (Garland et al., 2015; Gross, 2015). Although many ER interventions focus on reducing *dysregulation* (i.e., inability or difficulty regulating negative emotions; Sheppes et al., 2015) to address a range of mental health difficulties (e.g., trauma, substance use; Agathos et al., 2025; Aldao et al., 2015) emerging research highlights the importance of regulating (e.g., uplifting, boosting) positive emotions. Specifically, strategies that enhance or sustain positive emotions can impact how individuals cope with and recover from distress (Colombo et al., 2024; Zander-Schellenberg et al., 2020).

Overall, identifying interventions that enhance effective ER, including downregulating negative and upregulating positive emotions, is critical for helping transitional youth manage adversity (Brooks and Turner, 2026). Furthermore, behavioral health professionals require stronger empirical guidance on how to implement accessible, evidence-based tools to improve psychological outcomes (Venkatesan et al., 2026). Thus, the purpose of the current study was to evaluate how different facets (valence, tempo) of self-selected music listening upregulate positive emotions and downregulate negative emotions following exposure to a social stressor.

### Positive psychological theory

Championed by Seligman and Csikszentmihalyi (2000), positive psychology, the scientific study of how positive experiences, traits, and institutions improve life satisfaction and protect against declines in mental health, remains a central framework for understanding how well-being outcomes are explained and achieved through applied action (Carr et al., 2024). A core aim of positive psychology is to establish theoretically grounded, empirically supported models that identify the key building blocks for well-being (Donaldson et al., 2021). Within this aim, emotions and their regulation are critical as they play a central role in formulating, maintaining, and expanding well-being across domains such as health, community, work-life balance, and personal growth (Blasco-Belled et al., 2025; Wang et al., 2025). Newer models of positive psychology emphasize the interplay between positive and negative emotions in fostering well-being (Wong, 2019), reflecting a stark shift in philosophy. Earlier approaches viewed positive and negative emotions as separate, often focusing primarily on cultivating positive experiences in exclusively neutral or positive spaces (Wong, 2019). However, growing evidence suggests that well-being is present within adversity (e.g., post-traumatic growth; Elderton et al., 2017), underscoring the need for more integrative models that recognize the complementary roles of both positive and negative emotions in promoting well-being (Ryff, 2022).

### Positive and negative emotions

Emotions are complex reaction patterns with unique behavioral, physiological, and subjective components (Gross et al., 2006). They are often characterized by their functional elements, particularly in how they promote adaptive behaviors and increase well-being (Hartmann, 2024). To distinguish among different emotions, researchers commonly categorize emotions into negative and positive systems (Cacioppo and Berntson, 1999). Although these systems differ across key dimensions such as intensity, frequency, valence, and the processes by which they are generated (Gross and Jazaieri, 2014), evidence suggests they are not opposite ends on the same continuum (Iasiello et al., 2020). Positive emotions are commonly characterized by high valence states (Fredrickson, 2013), including happiness, joy, hope, and contentment. While their full range of benefits continue to be explored, they are strongly linked to reward processing (Craske et al., 2016). Notably, positive emotions help individuals savor future positive events (reward anticipation), recognize positive aspects of an experience (reward consumption), and take responsibility for positive outcomes (reward learning). Collectively, these processes are associated with reduced rates of mood- and anxiety-based symptoms (Craske et al., 2019). In contrast, negative emotions are low valence states commonly associated with states such as fear, sadness, frustration, and irritation (Fredrickson, 2013). Although often perceived as undesirable, negative emotions play an essential role in adaptive functioning (Ford et al., 2018). For example, emotions such as worry can signal potential threats and motivate protective or avoidant behaviors to increase safety (Sweeny and Dooley, 2017). However, if negative emotions are not regulated or become chronic, they are associated with declines in well-being (Aldao et al., 2015; Bradley et al., 2011).

While a significant portion of researchers evaluate positive and negative emotions outside the context of stress, both systems play a critical role in how individuals cope with adversity (Boemo et al., 2022; Colombo et al., 2024). Emotions arise when a stimulus, such as being delayed in traffic, interferes with goal achievement (Lazarus, 2006). Commonly, stressors (of varying degrees) lead to increased negative emotions and reduced positive emotions (Brouwers et al., 2013; Yaribeygi et al., 2017), which interferes with effective coping and reduces well-being (Ubillos-Landa and Puente-Martínez, 2026). Given these effects, the use of ER strategies is particularly important for enhancing coping effectiveness. Most notably, upregulating positive emotions and downregulating negative emotions are two valued approaches in managing and recovering from stress (Erdem et al., 2025; Klibert et al., 2022).

Upregulating positive emotions represents a promising target for reducing the impact of adverse events (Quoidbach et al., 2015). There is a growing level of support to confirm this position. At a basic level, individuals intentionally seek out positive emotions to reduce stress (Waugh, 2020). Research also suggests maximizing the generation of positive emotions reduces physiological reactivity underlying stress and strengthens the ability to make better coping decisions (Pressman et al., 2019). These effects also interact with broader protective processes; for example, increasing positive emotions can mitigate the affective consequences associated with social stressors, such as discrimination (Duker et al., 2022). Regarding resilience, emerging models suggest that boosting positive emotions buffers the effects of stress by activating mesostriatal reward networks in the brain, leading to better coping outcomes (Tabibnia, 2020).

Similarly, the downregulation of negative emotions plays a significant role in reducing the intensity and duration of stress responses. From a neuropsychological perspective, effective downregulation attenuates distress related activation in the amygdala, autonomic nervous, and hypothalamic pituitary adrenal systems (Tabibnia, 2020). One key downregulation strategy, acceptance, predicts lower negative emotional responses to standardized and daily stressors (Ford et al., 2018). Other downregulating approaches decrease negative emotions leading to enhanced coping (Speer et al., 2021) and stress recovery (Jentsch and Wolf, 2020) processes. Taken together, upregulation of positive emotions and downregulation of negative emotions are essential in addressing the deleterious effects of stress. However, further research is needed to identify and evaluate accessible and cost-efficient mechanisms to support ER capacities (Bettis et al., 2022).

### Music listening and emotion regulation

Listening to music holds promise as a non-invasive, personalized, accessible, and cost-effective approach to promote well-being (Torlak et al., 2025). As a result, the field of MER, which encompasses music listening behaviors, has grown substantially over the past two decades. From an ER perspective, music listening improves mood (Juslin and Vjastfall, 2008), reduces stress (Kenny and Faunce, 2004), and evokes positive emotional states such as relaxation (Zentner et al., 2008) to support a wide range of well-being outcomes. Depending on the listener’s emotional needs, music evokes an array of emotional states, including melancholy, rage, joy, euphoria (Gabrielsson, 2001) and incites distinct emotion-related physiological responses (e.g., zygomatic facial activity; Lundqvist et al., 2009). Meta-analytic studies confirm the effects of music listening on ER (Phillpotts et al., 2025; Wimbarti et al., 2025) demonstrating that music interventions have a moderate impact on ER (Peters et al., 2024) and are especially important in navigating everyday ER processes (Lee et al., 2026).

Given the emotional properties of music, people often use it to express or experience specific emotions (Corrigall and Schellenberg, 2015) or shift their emotions toward positive states (upregulation) or away from negative (downregulating) states (Baltazar and Saarikallio, 2016; Chong et al., 2024). Downregulating and upregulating properties of music listening are well established within the literature. Notably, people use music to revive (recover energy when down), divert (shift attention away from distressing feelings), discharge (release negative feelings), and find solace (search for peace and comfort with challenging environments; Saarikallio, 2008). These MER strategies are generally effective though their impact varies by context. For instance, positive music induction tasks lift emotional energy, specifically related to vigor and happiness states (Campbell et al., 2021), whereas other MER studies find music listening is successful in downregulating negative emotions, like loneliness (Martín et al., 2021). Despite this evidence, more experimental research is needed to address pressing gaps regarding the contexts by which music listening affects ER processes (Garrido et al., 2022; Leipold et al., 2025).

### Music listening in stressful contexts

Despite established links between musical engagement and emotional processes, many studies focus only on general mood improvement and associated health outcomes (Kenny and Faunce, 2004; Saarikallio and Erkkilä, 2007). Cross-sectional findings consistently associate music listening with adaptive coping strategies (Leipold et al., 2025), which suggests music interventions may be suited to offset the psychological and physiological consequences of stress. Furthermore, listening to music enhances relaxation and reduces physiological arousal after experiencing a stressful event (Yehuda, 2011). These findings are reinforced by meta-analytic studies, where music interventions effectively reduced heart rate, blood pressure, and stress-related hormones (de Witte et al., 2022). It is speculated that the beneficial effects of music on stress recovery are largely due to regulatory capacities; music listening promotes *emotion induction* (i.e., generating emotions), *emotional discharge* (i.e., the release of emotions), and *emotional solace* (i.e., comfort during times of emotional pain; Saarikallio and Erkkilä, 2007), all of which are important in managing stress-induced symptoms. However, few experimental studies pinpoint the ER mechanisms underlying how music listening supports better stress-related outcomes. Namely, do music listening interventions uplift positive emotions, downregulate negative emotions, or both? Overall, it is important to evaluate how people emotionally respond to music listening interventions after the experience of a stressor to better clarify ER effects.

### Self-selection in music listening

One of the more characteristic music listening behaviors is choosing music, or *self-selection.* Research indicates that self-selected music is more effective at regulating emotional responses, particularly negative emotions, than experimenter-selected music (Groarke et al., 2020; Groarke and Hogan, 2019). This is consistent with the predominant literature suggesting listeners self-select music based on specific needs or expectations, which influences ER processes (Batt-Rawden, 2010). For instance, listeners often seek out songs to which they have a personal connection when using music to cope with stress (Jiang et al., 2013). Furthermore, self-selected music may activate cognitive functions, like autobiographical memories (El Haj et al., 2012), to better shape and regulate listener emotions (Vuoskoski and Eerola, 2012). Psychologically, self-selected music is tied to other important resources. Namely, researchers pinpoint self-selected music as a mechanism to support a sense of agency (Saarikallio et al., 2020) and identity (Peck and Grealey, 2020) in navigating different life circumstances. Despite growing evidence for the beneficial effects of self-selection, there are no known studies outlining how self-selected music impacts positive emotions after a stressful event. Considering self-selected music demonstrates the potential to stimulate brain reward systems and affective networks (Venkatesan et al., 2026), there is clear potential for a causal relationship between self-selected music and positive emotional upregulation. Yet, researchers warn self-selection does not always align with psychological benefits (Larwood and Dingle, 2022); if self-selection reinforces negative mood states, then music choices may minimize upregulation benefits.

### Music qualities

Music’s effectiveness as an ER tool is neither uniform nor automatic. Its efficacy depends on specific musical attributes. For instance, listening to music without consideration for purpose, personal relevance, or its qualities reduces its emotional impact (Chin and Rickard, 2014; Weth et al., 2015). Regarding musical qualities, valence, level of pleasantness or positivity inherent within music, and arousal, level of energy (ranging from excitable to calm), are theorized to play key roles in supporting ER capacities underlying music listening (Koelsch et al., 2019; Russell, 1980). At a preliminary level, qualitative and correlational findings indicate musical valence is important in reducing distressing emotions and maintaining/improving positive emotions (McFerran et al., 2018; Papinczak et al., 2015); for instance, listening to pleasant and positive music is more likely to better regulate positive emotions. Comparable conclusions apply to arousal-related aspects like tempo, where high-tempo songs are believed to amplify and sustain energetic emotions (Liew et al., 2025; Thomas and Kumar, 2026). Interestingly, low tempo music can also be important in producing relaxed and calm emotional states (Saarikallio et al., 2021), which reinforce well-being. To accommodate the uniqueness of each of these findings, researchers encourage synergistic evaluations of how valence and arousal qualities intersect to downregulate and upregulate emotions (Chong et al., 2024).

A synthesis of available research suggests high valence music qualities may interact with high *and* low arousal qualities to impact ER. These two systems of music quality (high valence/high tempo and high valence/low tempo) are encapsulated into two different types of musical listening: empowering music and calming music. *Empowering music* is defined as music with high valence (positivity) and high arousal (high energy and tempo; Zentner et al., 2008), designed to promote subjective feelings of power (Hsu et al., 2015), vitality (Zentner et al., 2008), and self-confidence (Elvers et al., 2017). These emotional responses are key in uplifting positive emotions (Koelsch et al., 2019), reappraising negative experiences (Barbic et al., 2013), and promoting better stress recovery and coping processes (Choi, 2017). Moreover, empowering music is theorized to motivate individuals to engage in tasks and reduce distraction (Elvers et al., 2017; Koelsch et al., 2019), two important elements in managing stress (Hoyt et al., 2024). Overall, preliminary research suggests empowered music listening may serve as a valuable mechanism for diversifying how individuals regulate their emotions, particularly in stressful contexts.

Interestingly, the intersection between high valence and low arousal (tempo) qualities of songs may also offer some ER benefits to people. From a strength-based perspective, c*alming music* is defined as music with higher valence (positivity), low arousal (low energy and slow tempo; Lilley et al., 2014), and repetitive rhythm (Knight and Rickard, 2001), designed to promote subjective feelings of relaxation, soothing, and peace (Bigliassi et al., 2015). Musical qualities centering relaxation and calmness are hypothesized to be a core element of MER (Van Goethem and Sloboda, 2011). However, only a handful of studies provide evidence for ER capacities underlying calming and relaxing music. Namely, relaxing musical sounds decrease negative emotions and depressed mood, consistent with the basic tenets of downregulation principles (Witten et al., 2023). From an upregulation perspective, few studies report causal evidence for how calming and relaxing music uplift positive emotions. However, low tempo music accompanying mindfulness-based activities does increase more low-arousal positive emotions, including serenity and peace (Tang et al., 2024). Researchers also note evidence for the anxiolytic effects of calming music in the context of stressful events (e.g., surgical procedures; Kühlmann et al., 2018). Similarly, low tempo and relaxing music support greater recovery after the experience of cognitive-based stress induction tasks (Baccarani et al., 2023). Other studies indicate relaxing and calming music reduces negative emotions (i.e., anger) in response to an anger arousal induction task (Zhang et al., 2024). Overall, different combinations of musical qualities (valence, tempo) warrant further evaluation in determining how MER interventions upregulate and downregulate emotions after the experience of stress.

### Current study

Psychological research demonstrates a strong empirical connection between music and emotions. Music listening is a viable intervention given its wide accessibility, low cost, and relevance across cultures (Juslin and Vjastfall, 2008; Saarikallio and Erkkilä, 2007). Despite these factors, further research is needed to evaluate how distinct categories of self-selected music help individuals regulate emotions (downregulate, upregulate), especially after a stressful event. Broadly, this study aimed to explore music listening as a potential MER tool. We investigated the effects of self-selected music (empowering and calming playlists) on emotions after a stress induction task using an experimental design. Considering the current literature, we made dual hypotheses regarding the effects of different types of music listening interventions on ER outcomes:

1. Listening to self-selected *empowering music* (high valence, high tempo) will decrease negative emotions and increase positive emotions after a stressful experience.
2. Listening to self-selected *calming music* (high valence, low tempo) will decrease negative emotions and increase positive emotions after a stressful experience.

At the exploratory level, understanding which self-selected music types—empowering or calming—best regulate emotions is vital. The lack of clear guidance from the literature on how different music listening interventions impact emotional responses after stress prevents the formulation of specific hypotheses and limits theory generation regarding how positive psychology and music can interact to promote better ER outcomes.

## Materials and method

### Research design

A multi-phase research design was employed to examine the effects of self-selected music on emotions, following a stress induction task. In Phase 1, a set of cross-sectional self-report questionnaires screened participants’ music preferences. These data were used to formulate playlists for participants selected to participate in Phase 2. Using a separate experimental design, Phase 2 randomly assigned participants into one of three intervention conditions (empowering music group, calming music group, or control group) after receiving a stress induction task. The experiment tracked participants’ self-rated emotional states (positive and negative) across three time points (baseline, post-stress induction, post-intervention).

### Phase 1 research procedures

**Figure 1:**
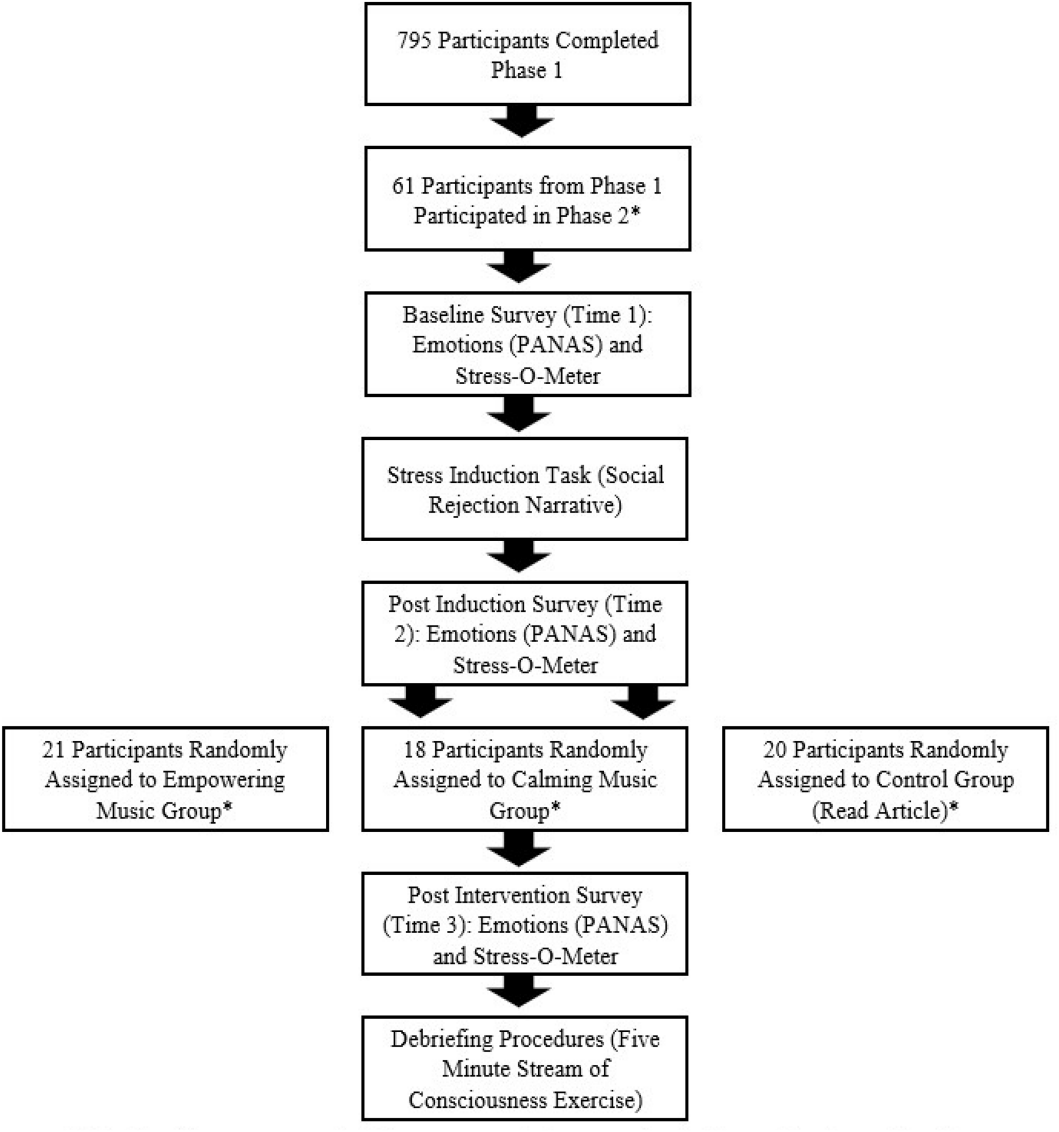
Study procedures flow chart.

A flow chart of the study procedures is presented in Figure 1. Institutional Review Board approval was obtained before data collection. During Phase 1, screening data were collected through a Qualtrics survey. The purpose of Phase 1 was to collect preferred empowering and calming songs for Phase 2. Data were collected regarding participants’ demographic information and music preferences. Participants rated preferred songs on level of familiarity, preference, induced feelings of empowerment, and induced feelings of calm. Participants spent around 50 min completing the screening surveys, after which they could opt into Phase 2. Participants received extra credit for psychology courses as compensation. Inclusionary criteria based on deviant response patterns were applied to ensure data quality. To be considered in the final sample, participants needed to express a desire to participate in Phase 2 and complete at least 90% of the survey items in Phase 1.

### Phase 1 participants

Undergraduate students from a large southeastern university in the United States (US) were recruited to participate. Inclusionary criteria included being 18 years of age or older (the age of consent in the US). In addition, participants needed to express an interest in participating in a music-based research project. There were no exclusionary criteria associated with this phase of the study. In total, 795 participants aged 18 to 54, with an average age of 20.17 years (*SD* = 4.24), completed the research protocol. Of these participants, 64.4% identified as white, cisgender women.

### Phase 1 measures

In Phase 1, participants completed a sociodemographic form, music listening preferences questionnaire (Music Rating Form), and the Short Test of Music Preferences (STOMP). They were also asked to rate a playlist with songs from their preferred genre on levels of induced calming and empowering feelings. All measures were administered via a Qualtrics survey.

#### Short test of music preferences

The STOMP is a brief measure of music genre preferences requiring participants to listen to brief music excerpts and rate their level of preference using a 7-point rating scale with endpoints at 1 (Not at All) and 7 (A Great Deal). The STOMP comprises 14 music genres: alternative, blues, classical, country, electronica/dance, folk, heavy metal, rap/hip-hop, jazz, pop, religious, rock, soul/funk, and soundtracks. A fifteenth genre, Latin, was added by the researchers for this study. Latin music is rooted in *bolero*, *salsa*, and *tango* genres and is rising in popularity in the US (Kennon, 2018). The purpose of administering the STOMP was to gather data on participants’ music genre preferences for playlist creation (Rentfrow and Gosling, 2003).

#### Music rating form

To evaluate participants’ experience of empowerment and calm related to music in their preferred genre, we constructed a music rating form. Music rating forms were idiosyncratically built based on how participants responded to the STOMP. If a participant rated a genre as “Like Very Much” or “Strongly Like,” they received a playlist for that genre to evaluate. For instance, if a participant rated Rock music as *“strongly like,”* Alternative music as “*strongly like,”* and Latin music as “very much like*”*, they were asked to evaluate three separate playlists associated with those genres of music. Each genre-specific playlist consisted of 15 songs. The researchers developed each genre-specific playlist using the Spotify Application Program Interface (API) *Sort Your Music.* Songs were selected based on an equitable dispersion of song characteristics (i.e., valence and tempo). Importantly, for each genre playlist, songs were chosen to represent empowering (*n* = 5), calming (*n* = 5), and ambiguous (*n* = 5) features. How songs fit into each of these groups was guided by Thayer’s (1989) arousal–valence framework, which conceptualizes emotional experiences as a function of valence and arousal.

**Figure 2:**
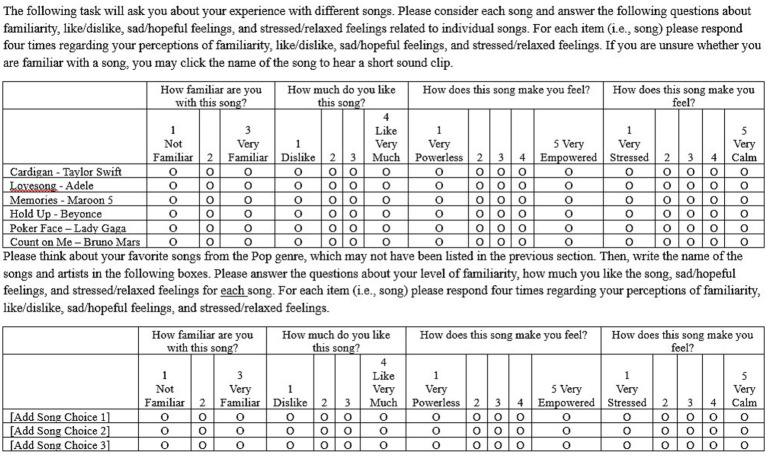
Pop genre-specific playlist example from the music rating form.

Empowering songs (high tempo, high valence; Zentner et al., 2008) were defined as those rated on the 50th percentile or above on energy and valence, whereas calming songs (low energy, moderately high valence; Lilley et al., 2014) were defined as those rated lower than the 50th percentile on energy and as high as the 70th percentile on valence.^1^ Finally, ambiguous songs (neither high nor low on energy or valence) were defined as those rated between the 40th and 60th percentile on energy and between the 35th and 65th percentile on valence. All selected songs were considered preferred and familiar, rated in the top 100 of each genre. All song titles were hyperlinked to file recordings of the songs to allow participants to increase familiarity with the listed music, see Figure 2. Participants were also given the option to write in up to three self-selected “favorite songs” that were not included in the genre-based sample playlists, see Figure 2.

For all songs listed in each participant’s preferred genre-specific playlist, they were asked to rate them on levels of familiarity (using a three-point scale from 1 = unfamiliar to 3 = very familiar), preference (using a four-point scales from 1 = dislike to 4 = like very much), and induced “empowering” (using a five-point scale from 1 = “feel very powerless” to 5 = “feel very empowered”) and “calming” (using a five-point scale from 1 = “feel very stressed” to 5 = “feel very calm”) feelings. Any additionally identified favored songs were written into the survey by the participants and evaluated using these same metrics noted for Spotify playlist songs. Approximately, 77% of participants in Phase 2 wrote favored songs into the survey. These songs were prioritized in participant playlists.

#### Invitation questionnaire

At the end of the survey, we asked participants to evaluate their interest in participating in the second phase of the research study. After offering a brief description of Phase 2 procedures, we asked participants if they had any interest in completing Phase 2. If they acknowledged interest, we asked them to provide their name and preferred means of contact.

### Phase 2 research procedures

The study’s Phase 2 procedures are listed in Figure 1. Interested participants from Phase 1 were contacted via email to schedule an in-person session held in the researcher’s lab. Eligibility for Phase 2 required participants to be at least 18 years old, provided viable contact information, reported preferred songs in the Phase 1 screener, and completed at least 90% of the Phase 1 survey. There were no exclusionary criteria for this portion of the study. Approximately 85% (71 out of 84) of recruited participants arrived at the lab during their scheduled time. The lab was constructed to mimic the form and function of a therapy room. It contained comfortable seating with a wall mounted two-way mirror. Researchers provided instructions to participants before every task and left the lab space to sit behind the two-way mirror while participants completed prescribed tasks. During task completion, researchers were instructed to observe participant behaviors, particularly distress-related and inattentive behaviors (i.e., day dreaming, sleeping, fidgeting) using a brief record form. If researchers observed any distress-related behaviors (e.g., crying), they were instructed to immediately end the study. No distress-related behaviors were observed; thus, the study was not terminated prematurely for any participant.

After sessions were scheduled with participants, the researchers constructed idiosyncratic playlists (empowering and calming) for each participant using the Spotify application. Playlists were developed using the data collected in Phase 1. Playlists’ songs were matched with participants’ musical interests. Namely, all songs within the playlists were consistent within the preferred musical genre of participants. In addition, participants’ favored songs (i.e., those specifically written in on the Music Rating Form) were prioritized for inclusion in the playlists. Selected playlist songs were all rated highly (a score of four or five) by participants as empowering or calming on the Music Rating From, depending on the playlist developed. One final consideration for inclusion into the playlist was song length. Each playlist developed allowed for approximately 15 minutes of listening time, plus or minus 30 s. In total, all playlists created included three to five songs (depending on the total time of each song on the playlist) situated within the participant’s preferred genres of music, acknowledged as preferred or favored, and rated highly as empowering or calming (depending on the list created). Empowering and calming playlists were downloaded onto the computer before participants arrived. The determination of what playlist was to be used during administration of the study was dependent upon random assignment.

When participants entered the lab, they were ushered into a comfortable seat, where they read and signed an informed consent document. After signing the consent form, participants completed a baseline series of measures on a roll-away desktop computer. Next, on the same roll-away computer, participants completed the social rejection stress induction task, followed by a series of post-induction task measures. Participants were then randomly assigned to one of three conditions: empowering music group, calming music group, or control group. All elements of the conditions were administered through the computer. Participants received over-the-ear Bose Quietcomfort 35 Series II noise-canceling headphones to listen to their playlist. After completing the assigned intervention condition, participants completed a final set of post-intervention measures. Finally, participants engaged in an active debriefing procedure consisting of a five-minute stream-of-consciousness mindfulness exercise (Wilson and DuFrene, 2009) to mitigate any residual distress. Participants were also provided with a debriefing form that included free and low-cost counseling resources. Compensation for Phase 2 participation was a $10 Amazon e-gift card.

### Phase 2 participants

Participants for Phase 2 consisted of individuals who acknowledged interest in continued participation in the project as noted in the Invitation Questionnaire in Phase 1. Those who met criteria to participate were randomized into a contact order list. The researchers contacted each eligible participant, according to the contact order list, to schedule an in-person meeting to complete Phase 2. Eligible participants were contacted until the sample was filled or no other eligible participants were identified.

Regarding statistical power, we used a power calculator to determine the sample size for Phase 2. We set the following parameters, significance level (*α*) = 0.05, statistical power (1 – *β*) = 0.8, and effect size (*d*) = 0.5, to determine the minimal number of participants needed to detect large effects. The calculator indicated a total sample size of 42 was needed to detect large effects. To increase our ability to detect medium effects, we re-ran the analysis with a lower effect size (*d*) = 0.35. Results indicated a sample size of 84 was needed to detect medium effects.

Due to limits related to scheduling and incentive funds, we only ran 71 participants in Phase 2. However, to ensure data quality, we set *a priori* criteria for acceptable data. Importantly, we removed data if participants provided insufficient responses (e.g., left 10% of the survey items blank) or demonstrated concentration/motivation difficulties (e.g., sleeping) during Phase 2 as noted by behavioral observations. Ten participants were removed from the data analysis due to incomplete surveys or appearing distracted during the experiment. Thus, a total of 61 were retained in the final sample for Phase 2. Participants ranged in age from 18 to 27 years, with an average age of 19.14 years (SD = 1.74). Other socio-demographic information for Phase 2 is provided in Table 1.

**Table 1:** Demographic characteristics of participants (*n* = 61).

Item	Category	Frequency (*f*)	Percentage (%)
Gender	Cisgender men	15	24.6
	Cisgender women	45	73.8
	Transgender	1	1.6
Age (years)	18–20	52	88.2
	21–25	5	8.2
	26+	2	3.2
Ethnicity	White/Non-Hispanic	42	68.9
	African American/Black	12	19.7
	Hispanic/Latine	4	6.6
	Asian American	1	1.6
	Other	2	3.3
Socioeconomic status (SES)	Low SES	4	6.6
	Low-Middle SES	13	21.3
	Middle SES	26	42.6
	Middle-High SES	18	29.5

### Phase 2 measures

Measures of emotions and stress were collected three times during Phase 2: baseline, post-induction task, and post-intervention. All measures were administered in a Qualtrics survey.

#### Positive and negative affect schedule

Positive and negative emotions scores were obtained through three administrations (baseline, post-induction, post-intervention) of the PANAS. The PANAS (Watson et al., 1988) is a 20-item self-report measure assessing emotional states using two scales: Positive Affect (PANAS-PA) and Negative Affect (PANAS-NA), each with items rated on a 5-point rating scale from 1 (Very Slightly or Not at All) to 5 (Extremely). High scores on PANAS-PA indicate satisfaction and happiness, while high PANAS-NA scores suggest distress and irritability. PANAS-PA and PANAS-NA scores served as the dependent variables within the current study. The PANAS demonstrates good psychometric properties (Crawford and Henry, 2004). Within the current study, the PANAS demonstrated solid internal consistency (PANAS-PA α = 0.84 to 0.93; PANAS-NA α = 0.79 to 0.84).

#### Stress-O-meter

The SOM (Keegan et al., 2015) is a one-item measure of state stress ranging from 0 (*No Stress*) to 100 (*Extreme Stress*). The SOM demonstrates good psychometric properties (Keegan et al., 2015). The SOM was administered three times during this phase of the study (baseline, post-induction, post-intervention).

### Phase 2 stress induction task

#### Construction

We constructed an induction task designed to elicit mild to moderate reports of stress consistent with a social-evaluative hassle or conflict. To accomplish this goal, we referenced commonly used induction tasks, like the Trier Social Stress Test (TSST; Kirschbaum et al., 1993), effective in eliciting mild forms of upset within transitional youth samples (Klibert et al., 2022). However, we augmented the structure and orientation of these types of induction tasks to center more developmentally relevant conflicts and concerns, specifically social rejection. This decision was made because transitional youth experience at least one perceived rejection per day (Xie et al., 2023), therefore recollections of such events may be easier for them to process and describe. Within our lab, we pilot tested the effects of similar (length, content area, format) induction tasks with transitional youth samples and results indicated the inductions produced transient mild to moderate levels of stress. Moreover, the induction task was reviewed by intervention specialists as part of the IRB process.

#### Administration

After baseline measures were administered, researchers informed participants they would be completing a reflection and journaling task. Specifically, researchers instructed each participant to consider a recent instance where they felt rejected. Once participants acknowledged they could describe a situation where they felt rejected, the researcher asked them to write about their experience in a narrative format on a roll-away desktop computer. Participants were instructed to highlight as many details and related emotions as possible in a 400-word journaling exercise. After participants acknowledged their understanding of the task, the researcher left the room so participants could write in private. Once participants completed the induction task, researchers instructed them to save the document in an encrypted and anonymous file on the computer. The document was used to evaluate how participants engaged with the writing task and followed instructions. All participants were able to write at least 400 words and completed the task in just under 15 min.

### Phase 2 music interventions

#### Control group

Following the stress induction task, participants in the control group engaged in a low-emotional activity: reading a pre-screened, neutrally toned five-page article on music theory for 15 min. They were instructed to read mindfully, focus on details, and complete the article in its entirety. This approach aimed to elicit minimal emotional stimulation for comparison with other conditions. Below is the language used to instruct participants on how to participate in the control group exercise.

“Not sure if this is noteworthy, but this quote seems formated differently. If you look on the next page, the quote under the Empowering music group is a little different in terms of font color and indention. Again, not sure if this is something that needs to be corrected, but I thought I would bring it up. The article will be available to you on this computer screen. Please use the mouse to navigate the pages of the article”.

#### Empowering music group

Participants in this group received personalized empowering music playlists. Curated beforehand, these playlists incorporated participants’ preferred music genres and their self-identified empowering songs within those genres, as outlined in their screening surveys. All included empowering songs were rated as highly preferred or as a favored (written in) song. Specifically, all songs on the playlist were rated as Like or Like Very Much (preference) and Empowered or Very Empowered (empowering) on participants’ Music Rating Form. Participants wore over-the-ear headphones and were instructed to listen mindfully to their 15-min (~3–5 song) Spotify playlist streamed via a desktop computer. Participants controlled volume independently via an external dial and each playlist concluded naturally at the end of a song. Below is the language used to instruct participants on how to listen mindfully to their playlist.

“Please mindfully listen to the following playlist. Remember to pay attention to the music, notice all the sounds, echoes, and vibrations. When you’re ready, place the headphones over your ears and press the start button on the audio player.”

#### Calming music group

The protocol and instructions for this group are the same as for the Empowering Music group except with calming music content derived from participants’ screening surveys. All included calming songs were rated as highly preferred or as a favored (written in) song. Specifically, all songs on the playlist were rated as Like or Like Very Much (preference) and Calm or Very Calm (calming) on participants’ Music Rating Form. The language used to instruct participants on how to listen mindfully to their playlist was the same for the Empowering Music Group.

## Results

### Data processing

All analyses were conducted via SPSS. Before running the analyses, data were checked for outliers and violations of normality. Other assumptions (e.g., homogeneity of variance) were tested before specific analyses were conducted. Next, we ran a series of analyses to determine if the stress induction task was effective in its purpose. Finally, we ran a series of factorial ANCOVAs to determine if time interacted with intervention condition to explain variation in positive and negative emotion scores consistent with our hypotheses.

### Normal distribution and outliers

We evaluated markers of skewness and kurtosis to determine if participants reported normally distributed scores on baseline measures of stress, positive emotions, and negative emotions. After reviewing histograms, there were clear patterns of non-normality with stress and negative emotions scores. Stress, *D*(59) = 0.21, *p* < 0.01, and negative emotion, *D*(59) = 0.22, *p* < 0.01, scores were depicted as a positive skewed pattern and were determined to violate the assumption of normality. Positive emotion scores were normally distributed, *D*(59) = 0.08, *p* > 0.05. While non-normal distributions are not ideal, the *t-test* and the *F* statistic are robust enough to manage violations as long as the sample size is not extremely low and outliers are sufficiently managed (Blanca et al., 2017). Next, we evaluated all data points in efforts to detect extreme outliers. Because some of the variables were non-normally distributed, we employed the median absolute deviation (MAD; Leys et al., 2013) method for outlier detection. Specifically, we estimated the median for all scores in a set, then calculated the absolute difference from the median for each individual score. We then estimated Tukey’s fence, constructing lower and upper boundaries on the interquartile range. Scores that fell outside of lower and upper boundaries were pinpointed as outliers. We detected two outliers, scores that fell between −3 (lower boundary) and 36 (upper boundary), on the Negative Emotion score at post-induction. Notably, two participants violated the upper boundary criteria with scores of 37 and 38. These two outliers were removed from further consideration in any subsequent analysis.

After removing outliers from the study, there was a small imbalance among the sample sizes for each intervention condition group. The final sample size for each group was as follows: empowered listening group (*n* = 21), calming listening group (*n* = 18), control group (*n* = 20). Largely, the imbalance stems from participants violating attention checks (*n* = 10), failing to show up for their scheduled session (*n* = 9), and reporting in highly discrepant ways (outliers, *n* = 2). Subsequent analyses evaluated violations of homogeneity of variance to determine whether these imbalances negatively affected our ability to accurately interpret the data. To address concerns regarding multiple comparisons, Bonferroni corrections were employed in all between-subject analyses conducted within the study.

### Manipulation check

To determine whether the stress induction task was effective, we conducted a repeated measures *t*-test between Time 1 (baseline) stress and Time 2 (post-induction) stress. Results indicated participants, regardless of intervention condition group, reported higher levels of stress at Time 2 (*M* = 39.68, SD = 24.04) when compared to Time 1 (*M* = 28.58, SD = 25.06), *t* (58) = −4.55, *p* < 0.001, *d* = −0.592, which represents a moderate effect size. To evaluate any group differences in changes in stress scores from Time 1 to Time 2, we used a one-way between-groups Analysis of Variance (ANOVA)^2^ to evaluate stress change scores between Time 1 and Time 2. That is, Time 2 scores were subtracted from Time 1 scores, and change in stress scores served as the dependent variable. Levene’ test for equality of variances indicated that the data did not violate the homogeneity of variance assumption, *F* (2, 56) = 1.41, *p* = 0.252. The results revealed no significant differences between intervention condition groups, *F* (2, 56) = 0.4, *p* = 0.643, *_partial_η^2^* = 0.01. See Table 2 for the means and standard deviations for Time 1 and Time 2 stress scores.

**Table 2:** Descriptive statistics for change in stress scores from baseline to post-induction by group.

Dependent variable	Independent variable (group)	n*	Mean	Standard deviation
Stress	Empowering music group	21	−8.28	18.17
	Calming music group	18	−11.78	14.33
	Control group	20	−13.45	22.94

*Two individuals were removed from the control group as outliers.

### Primary analysis

A 2 × 3 (Time x Intervention Condition) mixed-factorial Analysis of Covariance (ANCOVA) with baseline scores as the covariate was used to examine variation in negative emotion scores. Regarding the within-subject effect, results revealed a non-significant main effect for time *F* (1, 55) = 0.17, *p* = 0.68, *_partial_η^2^* < 0.01. However, results detected a significant main effect for intervention condition group, *F* (2, 55) = 6.03, *p* < 0.01, *_partial_η^2^* = 0.18. Bonferroni post-hoc tests were used to further examine group differences. The empowering music intervention group reported significantly lower negative emotions than the control group at Time 2. This same pattern was detected at Time 3. There was also a significant difference between the control group and calming music group at Time 3, such that the control group reported higher negative emotions than the calming. There was no significant difference between the empowering music intervention group and calming music intervention group at Time 2 or Time 3 (see Table 3 for marginal means). The interaction between time and intervention condition was non-significant, *F* (2, 55) = 0.23, *p* = 0.8, *_partial_η^2^* < 0.01. Similarly, the interaction between the baseline covariate and the time factor was non-significant, *F* (1, 55) = 2.76, *p* = 0.1, *_partial_η^2^* = 0.05.

**Table 3:** Descriptive statistics for negative emotion scores at time (post-induction, post-intervention) across groups (empowering, calming, control).

Independent variable (time)	Independent variable (group)	n*	Mean	Standard deviation
Post-induction				
	Empowering music group	21	15.29	2.67
	Calming music group	18	16.56	5.73
	Control group	20	19.00	6.34
Post-intervention				
	Empowering music group	21	11.42	1.63
	Calming music group	18	12.00	2.72
	Control group	20	14.35	5.02

*Two individuals were removed from the control group as outliers.

**Figure 3:**
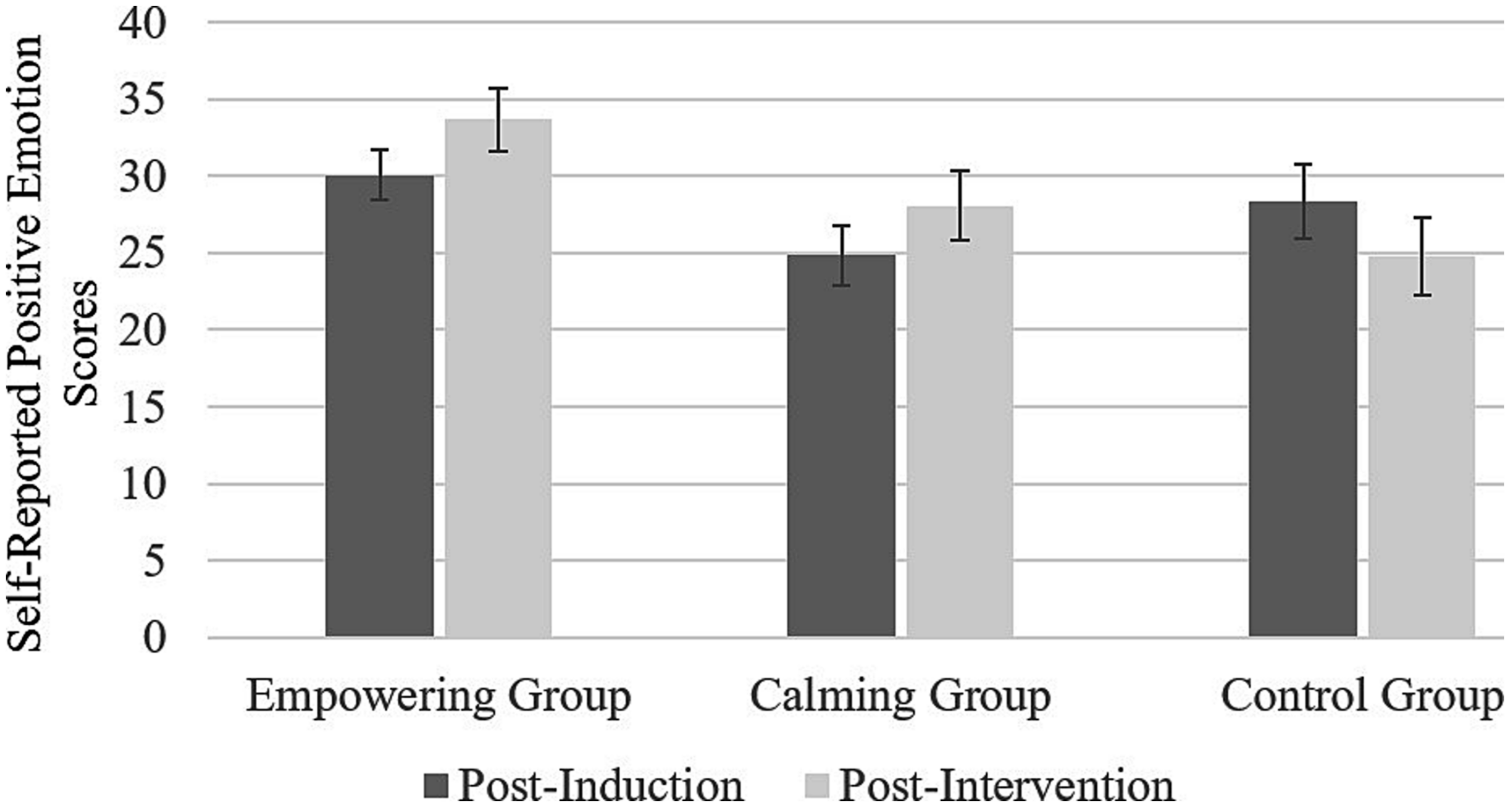
The interaction between time and condition on positive emotion scores.

Another 2 × 3 (Time x Intervention Condition) mixed-factorial Analysis of Covariance (ANCOVA) with baseline scores as the covariate was used to examine variation in positive emotion scores. Regarding the within-subjects effect, results revealed a non-significant main effect for time *F* (1, 55) = 0.12, *p* = 0.72, *_partial_η^2^* < 0.001. However, results detected a significant between-subjects main effect for intervention condition, *F* (2, 55) = 4.97, *p* < 0.01, *_partial_η^2^* = 0.15. Bonferroni post-hoc tests were used to further examine group differences. There was only one significant group difference detected. Specifically, the empowering music intervention group reported significantly greater positive emotions than the control group at Time 3 (see Table 4 for marginal means). Regarding interactions effects, results detected a non-significant interaction between the baseline covariate and the time factor, *F* (1, 55) = 0.01, *p* = 0.95, *_partial_η^2^* < 0.01. However, the main effects were qualified by a significant interaction effect between intervention condition group and time *F* (2, 55) = 8.48, *p* = < 0.001, *_partial_η^2^* = 0.24 (see Figure 3). To deconstruct the interaction further, we evaluated a series of paired-samples t-tests, particularly to evaluate how positive emotions scores changed from post-induction to post-intervention time points. Within the control group, members reported significantly less positive emotion scores at Time 3 (*M* = 28.35, SD = 10.7) compared to Time 2 (*M* = 28.35, SD = 10.7), *t* (19) = 3.06, *p* < 0.01, *d* = 0.685. The opposite pattern of change was detected in the other two groups. Namely, group members in the calming musical intervention group reported significantly higher positive emotions scores at Time 3 (*M* = 28.06, SD = 9.64) compared to Time 2 (*M* = 24.83, SD = 8.26), *t* (19) = −1.97, *p* < 0.05, *d* = −0.465, which is reflective of a small to moderate effect. Similarly, members of the empowering musical intervention group reported increases in positive emotion scores from Time 2 (*M* = 30.04, SD = 7.5) to Time 3 (*M* = 33.67, SD = 9.45), *t* (19) = −2.68, *p* < 0.01, *d* = −0.584, which is consistent with a moderate effect.

**Table 4:** Descriptive statistics for positive emotion scores at time (post-induction, post-intervention) across groups (empowering, calming, control).

Independent variable (time)	Independent variable (group)	n*	Mean	Standard deviation
Post-induction				
	Empowering music group	21	30.05	7.51
	Calming music group	18	24.83	8.26
	Control group	20	28.35	10.69
Post-intervention				
	Empowering music group	21	33.06	9.45
	Calming music group	18	28.06	9.64
	Control group	20	24.80	11.34

*Two individuals were removed from the control group as outliers.

## Discussion

Evidence suggests music listening promotes better ER capacities (Cook et al., 2019). However, more research is needed to clarify how characteristics of music listening impact downregulation of negative emotions and upregulation of positive emotions after a stressful experience. Using the work of Koelsch et al. (2019) as a theoretical backdrop, we investigated the impact of different music listening interventions on emotions after a social rejection induction task. Results highlight significant yet differential findings. Namely, specific types of self-selected music listening [empowering music (high valence, high arousal)] effectively upregulated positive emotions after a social rejection stressor. This finding provides some unique guidance in how musical listening interventions need to be structured to maximize the benefits of upregulation approaches after a social stressor.

Our findings highlight differential contexts by which music listening can upregulate positive emotions after a social rejection stressor. Individuals in the empowering music intervention reported greater positive emotions than those assigned to the control group at post-intervention. The size of this effect was moderate and consistent with current research suggesting high valence and high tempo music listening elicits positive emotions (Campbell et al., 2021; Koelsch et al., 2019). Importantly, this finding adds to the current literature. Namely, empowered music listening shows potential in upregulating positive emotions in unique contexts, like adversity. While negative emotions are predominant during stress, positive emotions do exist and it is important for them to be recognized and extended to support better well-being outcomes. Given our findings, targeted music listening strategies, like empowered music, appear to offer some unique opportunities to help individuals strengthen access to positive emotional states. However, more research is needed to clarify how empowered music listening supports emotional upregulation capacities during stress. For instance, our findings do not elucidate the mechanisms by which empowered music listening increases access to positive emotions after stress. ER researchers conclude there are several ways by which positive emotion regulation occurs, including cognitive reappraisal (Gross et al., 2006), emotional revival (Saarikallio, 2008), and mindfulness (Lalot et al., 2014). Moving forward, it will be important to determine if any of these specific upregulation strategies moderate the causal relationship between empowered music listening and positive emotions. It is also important to consider how empowered music listening is intertwined with autobiographical memories in generating and extending positive emotions during and after stressful events. Some research suggests high valence music listening taps into pleasant autobiographical memories, which reinforces the effectiveness of such interventions in promoting different well-being outcomes (El Haj et al., 2012). Overall, uncovering these pathways will provide a better understanding of how empowering music regulates positive emotions and how positive psychological interventions can be developed through musical frames.

It is also important to discuss the significant interaction effect within the context of clinical significance. Because of the moderate effect size by which empowered music listening contributed to increases in positive emotions, this finding meets basic level criteria for clinical significance (Brysbaert, 2019). Yet, it is important to refrain from making rigid determinations regarding clinical significance without more research. Thus, it will be important for future research to re-evaluate our findings by using mixed-method designs. Notably, adding qualitative components to the end of the study may help researchers evaluate if and how participants in empowering music listening groups experienced uplifts in positive emotions, which will support firmer claims of clinical significance. Similarly, conducting daily diary studies evaluating how participants use empowering music listening practices in managing daily life will be essential in corroborating the clinical utility of our findings.

While participants were afforded opportunities to have their favored songs curated within empowering music interventions, our study was unable to clarify the influence of self-selected music on the obtained effects. Most notably, we did not compare self-selected vs. experimenter selected musical listening conditions, which restricts our ability to discuss the potential benefits of self-selected music within the main contexts of the study. Moving forward, it will be important for researchers to evaluate the intersection between self-selected music and musical qualities (high valence, high tempo) and how it impacts positive emotion upregulation outcomes inside and outside the context of stress. This will require researchers to devise more dynamic methods of ensuring self-selected music meets basic musical criteria (valence, arousal; Koelsch et al., 2019) for empowered listening interventions. We believe our screening strategy (see Music Rating Form description) offers some unique platforms, like consideration for genre, self-reported ratings for targeted songs, inclusion of song familiarity/popularity, and prioritization of participant choice, in tackling this challenge. Yet, this process needs to be expanded to ensure each “favored song” designated as empowering meets musical quality criteria for empowered listening interventions. It is possible the use of AI technology (Wei and He, 2026) may be instrumental in evaluating how self-selected songs map onto musical criteria for empowering music interventions. Regardless, future research should prioritize examining how self-selected music is incorporated into targeted interventions, such as empowering and calming interventions.

Conversely, individuals assigned to the calming music intervention group did not report statistically significant increases in positive emotions post-intervention when compared to the control group. This finding is inconsistent with literature suggesting calming music generates pleasant emotions (Bigliassi et al., 2015). There are several potential explanations for this null effect. First, it is possible there was a mismatch between the type of stress-induced experience (i.e., social, emotional) and the type of musical intervention. Research suggests relaxing music improves mood and increases positive emotions after exposure to cognitively taxing tasks (e.g., arithmetic; Yamamoto et al., 2007) but less is known about how relaxing music impacts positive emotions after social stressful events (e.g., social rejection). Second, research often defines “relaxing music” with instrumental songs (i.e., classical music without lyrics; Chafin et al., 2004) and these specific genres tend to result in greater positive emotions among listeners (e.g., peacefulness; Zentner et al., 2008). No participants assigned to the calming music listening intervention group selected these types of music for inclusion in their playlist. Third, it might be more difficult for participants and researchers to identify clearly specific calming music (high valence, high tempo) across popular and familiar songs in different genres. For instance, in creating the Music Rating Form, we had difficulties pinpointing specific calming songs within the top 100 lists for all genres of music. It was easier to identify low tempo songs, but the valence of these songs was somewhat more ambiguous (less likely to be high) when compared to identifying other playlists (i.e., empowering [high valence, high tempo]). Given these points, it is important for research to develop more inclusive definitions of calming songs within the context of today’s landscape of music and determine how new conceptualizations of calming music contribute to the generation, maintenance, and expansion of positive emotional states.

Inconsistent with expectation, results failed to detect a significant time by intervention effect for negative emotions. This suggests individuals in the empowering and calming music listening groups did not report reduced levels of negative emotions post-intervention when compared to control group participants. This finding is at odds with prior research (Martín et al., 2021), highlighting the capacity of music listening to downregulate negative states/moods. It is possible the lack of a significant interaction effect was due to the unique context of the study. Specifically, most research identifying the downregulation effects of music listening were not evaluated in the context of a social stressor. Thus, the causal relationship between listening to music and reduced negative emotions may be conditional based on circumstance (ideal, neutral, adverse). However, one methodological limitation in our study may account for the non-significant effect. In evaluating mean scores across the duration of the study, interesting and concerning patterns emerged. Most notably, participants in all three groups reported declines in negative emotions from post-induction to post-intervention. This pattern was not consistent with the expected trajectory for control group participants, where we predicted negative emotions to linger (maintain) through the control group task. This suggests our control group activity (reading a music article) may not have evoked the expected neutral response intended. In fact, reading the article may have promoted different ER strategies (distraction; Sheppes and Meiran, 2008) leading to some unintended reductions in negative emotionality. Because of this, we cannot clearly determine whether empowering and calming listening behavior effectively downregulates negative emotions after a social stressor. Moving forward, it will be important for future research to re-evaluate our study using multiple control groups: an inactive control group (e.g., asking participants to sit and wait) and a more refined active control group (e.g., listening to a playlist with neutral valence and tempo). The use of such control groups might be better situated to capture natural fluctuations in negative emotions and isolate the clinical utility of empowering and calming music listening interventions.

It is also important to acknowledge the effects of our stress induction task in helping us accurately frame the results of the study. Our study’s stress induction task, a social rejection writing exercise, differs from typical music intervention studies that often use performance (e.g., mental math) or physiological (e.g., C02 stress test) methods. However, results indicated the task effectively increased stress levels, aligning with research supporting social writing tasks for stress elicitation (Kirschbaum et al., 1993; Moons and Shields, 2015). Despite these findings, it is important to recognize the potential limitations of our stress induction task and how those limitations impacted the primary results. Our induction task likely evoked idiosyncratic stress responses, which increases the potential of uncontrolled variance affecting how positive and negative emotions were managed through the study. For instance, some participants might have experienced a recent breakup or conflict within a close friendship prior to participating in the study. In turn, writing about these stressors as part of the induction task might have accelerated the intensity of negative emotions and decreased positive emotions, generating more barriers to effective ER processing for these individuals. Because of these concerns, future research should compare this method with other stress induction techniques to understand how they differently influence emotional responses in music studies.

### General limitations

The COVID-19 pandemic directly resulted in reduced power within this study. Specifically, the pandemic negatively impacted the recruitment and administration of the study to the optimal number of participants needed to detect a larger range of effect sizes. Furthermore, it is unclear whether participants in our study differed from those hesitant to join in-person experiments due to COVID-19 concerns. To validate our findings, it is essential to reassess the study’s questions now that the health risks of COVID-19 are effectively controlled. This study’s recruitment methods led to a purposive, availability-based sample of undergraduate students, limiting the findings’ generalizability to this group in the southeast US and not to broader community populations. The sample was also relatively homogenous in terms of ethnic identity, sexual orientation, and gender identity. Future researchers should confirm our findings with more demographically diverse samples. Additionally, data for this study were collected using self-report questionnaires, which are susceptible to social desirability bias and demand characteristics. It would be beneficial for future studies to use observational or psychophysiological measures to evaluate fluctuations in stress and emotions. It is also possible that the lab-based nature of the experiment negatively impacted the ecological validity of the findings. Future researchers might consider using apps or portable music players to collect data that is more reflective of real-life listening environments. Finally, we were unable to control for the effects of other musical characteristics (e.g., lyrics) on listener outcomes. Future studies may achieve more generalizable results by isolating the effects of diverse musical characteristics on different ER processes.

### Strengths of the study and general conclusions

This study uniquely demonstrated the effectiveness of empowering music over other music interventions in upregulating positive emotions following social stress. Our findings suggest creating short, personalized playlists of empowering songs can serve as a practical ER strategy to increase positive emotions, consistent with prevailing positive psychological theory. This approach underlines the potential clinical benefits of using music listening, particularly empowering music, to support ER processes. This perspective is valuable as it provides formulas for music therapists and applied positive psychologists to develop interventions that promote well-being using a highly accessible, cost-efficient, enjoyable, and personally meaningful tool (music listening). Our study underscores the critical role of evaluating the effects of musical listening during social forms of adversity. Our findings open new avenues for integrating empowering music into therapeutic frameworks, offering an accessible adjunct to traditional therapy.

## Data Availability

The original contributions presented in the study are included in the article/supplementary material, further inquiries can be directed to the corresponding author/s.

## Footnotes

1 It is important to note that very few songs on the top 100 list for each musical genre contained songs defined by high valence and low tempo. We chose songs with the highest valence possible, but these songs never reached above the 70 percentile. Because of this, some popular low tempo songs with moderate ratings for valence were included in the playlists.

2 Instead of running a factorial ANOVA with absolute stress scores as the dependent variable, we decided to create and evaluate change scores as the dependent variable in a one-way ANOVA. This decision was made in light of substantial variation in reported baseline stress scores across conditions, See Table 2. Using change scores, provides more accurate indications of whether the stress induction task produced comparable effects for individuals in different intervention conditions.

## References

[ref1] Agathos J. Yurtbasi M. O’Brien H. Putica A. (2025). The extended process model of emotion regulation in managing negative affect in posttraumatic stress disorder: a systematic review. Clin. Psychol. Sci. Pract. 32, 398–415. doi: 10.1037/cps0000296

[ref2] Aldao A. Sheppes G. Gross J. J. (2015). Emotion regulation flexibility. Cogn. Ther. Res. 39, 263–278. doi: 10.1007/s10608-014-9662-4

[ref3] Baccarani A. Donnadieu S. Pellissier S. Brochard R. (2023). Relaxing effects of music and odors on physiological recovery after cognitive stress and unexpected absence of multisensory benefit. Psychophysiology 60, e14251–e14216. doi: 10.1111/psyp.14251, 36700294

[ref4] Baltazar M. Saarikallio S. (2016). Toward a better understanding and conceptualization of affect self-regulation through music: a critical, integrative literature review. Psychol. Music 44, 1500–1521. doi: 10.1177/0305735616663313

[ref5] Barbic S. P. Bartlett S. J. Mayo N. E. (2013). Emotional vitality: concept of importance for rehabilitation. Arch. Phys. Med. Rehabil. 94, 1547–1554. doi: 10.1016/j.apmr.2012.11.045, 23262159

[ref6] Batt-Rawden K. B. (2010). The benefits of self-selected music on health and well-being. Arts Psychother. 37, 301–310. doi: 10.1016/j.aip.2010.05.005

[ref7] Bettis A. H. Burke T. A. Nesi J. Liu R. T. (2022). Digital technologies for emotion-regulation assessment and intervention: a conceptual review. Clin. Psychol. Sci. 10, 3–26. doi: 10.1177/21677026211011982, 35174006 PMC8846444

[ref8] Bigliassi M. Barreto-Silva V. Altimari L. R. Vandoni M. Codrons E. Buzzachera C. F. (2015). How motivational and calm music may affect the prefrontal cortex area and emotional responses: a functional near-infrared spectroscopy (fNIRS) study. Percept. Mot. Skills 120, 202–218. doi: 10.2466/27.24.PMS.120v12x5, 25650505

[ref9001] Blanca M. J. Alarcón R. Arnau J. Bono R. Bendayan R. (2017). Non-normal data: Is ANOVA still a valid option?. Psicothema, 29, 552–557. doi: 10.7334/psicothema2016.383 29048317

[ref9] Blasco-Belled A. Tejada-Gallardo C. Alsinet C. (2025). Positive psychology interventions can improve mental health for chronic pain patients: a systematic review and meta-analysis. Psychol. Health 40, 635–651. doi: 10.1080/08870446.2023.2250382, 37644768

[ref10] Boemo T. Nieto I. Vazquez C. Sanchez-Lopez A. (2022). Relations between emotion regulation strategies and affect in daily life: a systematic review and meta-analysis of studies using ecological momentary assessments. Neurosci. Biobehav. Rev. 139:104747. doi: 10.1016/j.neubiorev.2022.104747, 35716875

[ref11] Bradley B. DeFife J. A. Guarnaccia C. Phifer J. Fani N. Ressler K. J. . (2011). Emotion dysregulation and negative affect. J. Clin. Psychiatry 72, 685–691. doi: 10.4088/JCP.10m06409blu, 21658350 PMC4605672

[ref12] Brooks M. Turner M. J. (2026). A longitudinal model of emotion pathways to growth, depreciation, and health outcomes after life stress. Anxiety Stress Coping 39, 143–159. doi: 10.1080/10615806.2025.2558729, 40963154

[ref13] Brouwers C. Mommersteeg P. M. Nyklíček I. Pelle A. J. Westerhuis B. L. Szabó B. M. . (2013). Positive affect dimensions and their association with inflammatory biomarkers in patients with chronic heart failure. Biol. Psychol. 92, 220–226. doi: 10.1016/j.biopsycho.2012.10.002, 23085133

[ref14] Brysbaert M. (2019). How many participants do we have to include in properly powered experiments? A tutorial of power analysis with reference tables. J. Cogn. 2:16. doi: 10.5334/joc.72, 31517234 PMC6640316

[ref15] Cacioppo J. T. Berntson G. G. (1999). The affect system: architecture and operating characteristics. Curr. Dir. Psychol. Sci. 8, 133–137. doi: 10.1111/1467-8721.00031

[ref16] Campbell E. A. Berezina E. Gill C. M. H. D. (2021). The effects of music induction on mood and affect in an Asian context. Psychol. Music 49, 1132–1144. doi: 10.1177/0305735620928578

[ref17] Carr A. Finneran L. Boyd C. Shirey C. Canning C. Stafford O. . (2024). The evidence-base for positive psychology interventions: a mega-analysis of meta-analyses. J. Posit. Psychol. 19, 191–205. doi: 10.1080/17439760.2023.2168564

[ref18] Chafin S. Roy M. Gerin W. Christenfeld N. (2004). Music can facilitate blood pressure recovery from stress. Br. J. Health Psychol. 9, 393–403. doi: 10.1348/1359107041557020, 15296685

[ref19] Chi R. Tan J. (2025). *Neural Network Based Adaptive Framework for Music Emotion Regulation*. 2025 4th International Conference on Distributed Computing and Electrical Circuits and Electronics (ICDCECE), Distributed Computing and Electrical Circuits and Electronics (ICDCECE), 2025 4th International Conference On, pp. 1–7.

[ref20] Chin T. Rickard N. S. (2014). Emotion regulation strategy mediates both positive and negative relationships between music uses and well-being. Psychol. Music 42, 692–713. doi: 10.1177/030573561348991

[ref21] Choi G.-Y. (2017). Secondary traumatic stress and empowerment among social workers working with family violence or sexual assault survivors. J. Soc. Work. 17, 358–378. doi: 10.1177/1468017316640194

[ref22] Chong H. J. Kim H. J. Kim B. (2024). Scoping review on the use of music for emotion regulation. Behav. Sci. 14:793. doi: 10.3390/bs14090793, 39336008 PMC11428991

[ref23] Colombo D. Baños R. M. Desdentado L. Kleiboer A. Pavani J.-B. Wrzesien M. . (2024). Daily stress encounters: positive emotion upregulation and depressive symptoms. Emotion 24, 1403–1416. doi: 10.1037/emo0001362, 38512200

[ref24] Cook T. Roy A. R. K. Welker K. M. (2019). Music as an emotion regulation strategy: an examination of genres of music and their roles in emotion regulation. Psychol. Music 47, 144–154. doi: 10.1177/0305735617734627

[ref25] Corrigall K. A. Schellenberg E. G. (2015). “Liking music: Genres, Contextual Factors, and Individual Differences,” in Art, Aesthetics and the Brain, eds. Huston J. P. Nadal M. Mora F. Agnati L. F. Cela-Conde C. J. (Oxford: Oxford University Press).

[ref26] Craske M. G. Meuret A. E. Ritz T. Treanor M. Dour H. J. (2016). Treatment for anhedonia: a neuroscience driven approach. Depress. Anxiety (1091–4269) 33, 927–938. doi: 10.1002/da.22490, 27699943

[ref27] Craske M. G. Meuret A. E. Ritz T. Treanor M. Dour H. Rosenfield D. (2019). Positive affect treatment for depression and anxiety: a randomized clinical trial for a core feature of anhedonia. J. Consult. Clin. Psychol. 87, 457–471. doi: 10.1037/ccp0000396, 30998048

[ref28] Crawford J. R. Henry J. D. (2004). The positive and negative affect schedule (PANAS): construct validity, measurement properties, and normative data in a large, non-clinical sample. Br. J. Clin. Psychol. 43, 245–265. doi: 10.1348/0144665031752934 15333231

[ref29] De Witte M. Pinho A. D. S. Stams G.-J. Moonen X. Bos A. E. R. Van Hooren S. (2022). Music therapy for stress reduction: a systematic review and meta-analysis. Health Psychol. Rev. 16, 134–159. doi: 10.1080/17437199.2020.1846580, 33176590

[ref30] Donaldson S. I. Cabrera V. Gaffaney J. (2021). Following the science to generate well-being: using the highest-quality experimental evidence to design interventions. Front. Psychol. 12:352. doi: 10.3389/fpsyg.2021.739352, 34975628 PMC8715916

[ref31] Duker A. Green D. J. Onyeador I. N. Richeson J. A. (2022). Managing emotions in the face of discrimination: the differential effects of self-immersion, self-distanced reappraisal, and positive reappraisal. Emotion 22, 1435–1449. doi: 10.1037/emo0001001, 34591510

[ref32] El Haj M. Fasotti L. Allain P. (2012). The involuntary nature of music-evoked autobiographical memories in Alzheimer’s disease. Conscious. Cogn. 21, 238–246. doi: 10.1016/j.concog.2011.12.005, 22265372

[ref33] Elderton A. Berry A. Chan C. (2017). A systematic review of posttraumatic growth in survivors of interpersonal violence in adulthood. Trauma Violence Abuse 18, 223–236. doi: 10.1177/1524838015611672, 26459504

[ref34] Elvers P. Fischinger T. Steffens J. (2017). Music listening as self-enhancement: effects of empowering music on momentary explicit and implicit self-esteem. Psychol. Music 46, 307–325. doi: 10.1177/0305735617707354

[ref35] Erdem N. Roth G. Weinstein N. (2025). The role of integrative emotion regulation in adaptive coping and daily stress regulation. Stress Health 41, 1–16. doi: 10.1002/smi.70066 40727961

[ref36] Ford B. Q. Lam P. John O. P. Mauss I. B. (2018). The psychological health benefits of accepting negative emotions and thoughts: laboratory, diary, and longitudinal evidence. J. Pers. Soc. Psychol. 115, 1075–1092. doi: 10.1037/pspp0000157, 28703602 PMC5767148

[ref37] Fredrickson B. L. (2013). Positive emotions broaden and build. Adv. Exp. Soc. Psychol. 47, 1–53. doi: 10.1016/B978-0-12-407236-7.00001-2

[ref38] Gabrielsson A. (2001). Emotion perceived and emotion felt: same or different? Music. Sci. 5, 123–147. doi: 10.1177/10298649020050s105

[ref39] Garland E. L. Farb N. A. R Goldin P. Fredrickson B. L. (2015). Mindfulness broadens awareness and builds eudaimonic meaning: a process model of mindful positive emotion regulation. Psychol. Inq. 26, 293–314. doi: 10.1080/1047840X.2015.1064294, 27087765 PMC4826727

[ref40] Garrido S. Du Toit M. Meade T. (2022). Music listening and emotion regulation: young people’s perspectives on strategies, outcomes, and intervening factors. Psychomusicology 32, 7–14. doi: 10.1037/pmu0000285, 27371692

[ref41] Groarke J. M. Groarke A. Hogan M. J. Costello L. Lynch D. (2020). Does listening to music regulate negative affect in a stressful situation? Examining the effects of self-selected and researcher-selected music using both silent and active controls. Appl. Psychol. 12, 288–311. doi: 10.1111/aphw.12185, 31578781

[ref42] Groarke J. M. Hogan M. J. (2019). Listening to self-chosen music regulates induced negative affect for both younger and older adults. PLoS One 14, 1–19. doi: 10.1371/journal.pone.0218017 PMC6553776 31170224

[ref43] Gross J. J. (1998). The emerging field of emotion regulation: an integrative review. Rev. Gen. Psychol. 2, 271–299. doi: 10.1037/1089-2680.2.3.271

[ref44] Gross J. J. (2015). Emotion regulation: current status and future prospects. Psychol. Inq. 26, 1–26. doi: 10.1080/1047840x.2014.940781

[ref45] Gross J. J. Jazaieri H. (2014). Emotion, emotion regulation, and psychopathology: an affective science perspective. Clin. Psychol. Sci. 2, 387–401. doi: 10.1177/2167702614536164

[ref46] Gross J. J. Richards J. M. John O. P. (2006). “Emotion Regulation in Everyday Life,” in Emotion Regulation in Couples and Families: Pathways to Dysfunction and Health, eds. Snyder D. K. Simpson J. Hughes J. N. (Washington, D.C.: American Psychological Association).

[ref47] Hartmann K. (2024). Unlocking the language: key features of emotions. Acta Psychol. 251:104628. doi: 10.1016/j.actpsy.2024.104628, 39647453

[ref48] Healthy Minds Network. (2026). *The Healthy Minds Study: 2024–2025 Data Report*. Available online at: https://healthymindsnetwork.org/wp-content/uploads/2025/09/2024-2025_HMS-National-Data-Report_Student.pdf.

[ref49] Hoyt M. A. Llave K. Wang A. W.-T. Darabos K. Diaz K. G. Hoch M. . (2024). The utility of coping through emotional approach: a meta-analysis. Health Psychol. 43, 397–417. doi: 10.1037/hea0001364, 38330307 PMC11847609

[ref50] Hsu D. Y. Huang L. Nordgren L. F. Rucker D. D. Galinsky A. D. (2015). The music of power: perceptual and behavioral consequences of powerful music. Soc. Psychol. Personal. Sci. 6, 75–83. doi: 10.1177/1948550614542345

[ref51] Iasiello M. Van Agteren J. Cochrane E. M. (2020). Mental health and/or mental illness: a scoping review of the evidence and implications of the dual-continua model of mental health. Evid. Base 2020, 1–45. doi: 10.21307/eb-2020-00

[ref52] Jentsch V. L. Wolf O. T. (2020). The impact of emotion regulation on cardiovascular, neuroendocrine and psychological stress responses. Biol. Psychol. 154:107893. doi: 10.1016/j.biopsycho.2020.107893, 32437903

[ref53] Jiang J. Zhou L. Rickson D. Jiang C. (2013). The effects of sedative and stimulative music on stress reduction depend on music preference. Arts Psychother. 40, 201–205. doi: 10.1016/j.aip.2013.02.002

[ref54] Juslin P. N. Vjastfall D. (2008). Emotional responses to music: the need to consider underlying mechanisms. Behav. Brain Sci. 31, 751–621. doi: 10.1017/s0140525x08006079 18826699

[ref55] Keegan D. Byrne K. Cullen G. Doherty G. A. Dooley B. Mulcahy H. E. (2015). The Stressometer: a simple, valid, and responsive measure of psychological stress in inflammatory bowel disease patients. J. Crohn's Colitis 9, 881–885. doi: 10.1093/ecco-jcc/jjv120, 26221000

[ref56] Kennon C. (2018). Latin Music: The Evolution of an International Sound. New York: Greenhaven Publishing.

[ref57] Kenny D. T. Faunce G. (2004). The impact of group singing on mood, coping, and perceived pain in chronic pain patients attending a multidisciplinary pain clinic. J. Music. Ther. 41, 241–258. doi: 10.1093/jmt/41.3.241, 15327342

[ref58] Kirschbaum C. Pirke K.-M. Hellhammer D. H. (1993). The “Trier social stress test”: a tool for investigating psychobiological stress responses in a laboratory setting. Neuropsychobiology 28, 76–81. doi: 10.1159/000119004 8255414

[ref59] Klibert J. J. Sturz B. R. LeLeux-LaBarge K. Hatton A. Smalley K. B. Warren J. C. (2022). Savoring interventions increase positive emotions after a social-evaluative hassle. Front. Psychol. 13:1040. doi: 10.3389/fpsyg.2022.791040, 35386887 PMC8978832

[ref60] Knight W. E. Rickard N. S. (2001). Relaxing music prevents stress-induced increases in subjective anxiety, systolic blood pressure, and heart rate in healthy males and females. J. Music. Ther. 38, 254–272. doi: 10.1093/jmt/38.4.254, 11796077

[ref61] Koelsch S. Bashevkin T. Kristensen J. Tvedt T. Jentschke S. (2019). Heroic music stimulates empowering thoughts during mind-wandering. Sci. Rep. 9:10317. doi: 10.1038/s41598-019-46266-w, 31311967 PMC6635482

[ref62] Kühlmann A. Y. R. De Rooij A. Kroese L. F. van Dijk M. Hunink M. G. M. Jeekel J. (2018). Meta-analysis evaluating music interventions for anxiety and pain in surgery. J. Br. Surg. 105, 773–783. doi: 10.1002/bjs.10853, 29665028 PMC6175460

[ref63] Lalot F. Delplanque S. Sander D. (2014). Mindful regulation of positive emotions: a comparison with reappraisal and expressive suppression. Front. Psychol. 5:243. doi: 10.3389/fpsyg.2014.00243, 24715882 PMC3970027

[ref64] Larwood J. L. Dingle G. A. (2022). The effects of emotionally congruent sad music listening in young adults high in rumination. Psychol. Music 50, 218–229. doi: 10.1177/0305735620988793

[ref65] Lazarus R. S. (2006). Stress and Emotion: A new Synthesis. Berlin: Springer.

[ref66] Lee J. Ridder H. M. O. Lindvang C. (2026). Exploring the continuum of music listening for emotion regulation with university students: a meta-ethnography. Nord. J. Music. Ther. 35, 23–47. doi: 10.1080/08098131.2025.2488756

[ref67] Leipold B. Loidl B. Saalwirth C. Loepthien T. (2025). Emotion regulation through music listening, subjective stress, and problem-focused coping: longitudinal results. Musicae Sci. 29, 519–534. doi: 10.1177/10298649251321693

[ref68] Leys C. Ley C. Klein O. Bernard P. Licata L. (2013). Detecting outliers: do not use standard deviation around the mean, use absolute deviation around the median. J. Exp. Soc. Psychol. 49, 764–766. doi: 10.1016/j.jesp.2013.03.013

[ref69] Liew K. Koh A. H. Q. Fram N. R. Brown C. M. dela C. Lee L. . (2025). Groovin’ to the cultural beat: preferences for danceable music represent cultural affordances for high-arousal negative emotions. Psychol. Aesthet. Creat. Arts 19, 760–775. doi: 10.1037/aca0000599

[ref70] Lilley J. L. Oberle C. D. Thompson J. G. Jr. (2014). Effects of music and grade consequences on test anxiety and performance. Psychomusicology 24, 184–190. doi: 10.1037/pmu0000038

[ref71] Lundqvist L. O. Carlsson F. Hilmersson P. Juslin P. A. (2009). Emotional responses to music: experience, expression, and physiology. Psychol. Music 37, 61–90. doi: 10.1177/0305735607086048

[ref72] Martín J. C. Ortega-Sánchez D. Miguel I. N. Gil Martín G. M. (2021). Music as a factor associated with emotional self-regulation: a study on its relationship to age during COVID-19 lockdown in Spain. Heliyon 7:e06274. doi: 10.1016/j.heliyon.2021.e06274, 33665439 PMC7907216

[ref73] McFerran K. S. Hense C. Koike A. Rickwood D. (2018). Intentional music use to reduce psychological distress in adolescents accessing primary mental health care. Clin. Child Psychol. Psychiatry 23, 567–581. doi: 10.1177/1359104518767231, 29669441

[ref74] Moons W. G. Shields G. S. (2015). Anxiety, not anger, induces inflammatory activity: an avoidance/approach model of immune system activation. Emotion 15, 463–476. doi: 10.1037/emo0000055 26053247

[ref75] Papinczak Z. E. Dingle G. A. Stoyanov S. R. Hides L. Zelenko O. (2015). Young people’s uses of music for well-being. J. Youth Stud. 18, 1119–1134. doi: 10.1080/13676261.2015.1020935

[ref76] Peck L. S. L. Grealey P. (2020). Autobiographical significance of meaningful musical experiences: reflections on youth and identity. Music Sci. 3:4221. doi: 10.1177/2059204320974221

[ref77] Peters V. Bissonnette J. Nadeau D. Gauthier-Légaré A. Noël M.-A. (2024). The impact of musicking on emotion regulation: a systematic review and meta-analysis. Psychol. Music 52, 548–568. doi: 10.1177/0305735623121236, 39297022 PMC11405141

[ref78] Phillpotts E. Fourlemadis N. Petrini K. (2025). Music listening for wellbeing and coping during times of crisis. Music Med. 17, 132–157. doi: 10.47513/mmd.v17i3.989

[ref79] Pressman S. D. Jenkins B. N. Moskowitz J. T. (2019). Positive affect and health: what do we know and where next should we go? Annu. Rev. Psychol. 70, 627–650. doi: 10.1146/annurev-psych-010418-102955, 30260746

[ref80] Quoidbach J. Mikolajczak M. Gross J. J. (2015). Positive interventions: an emotion regulation perspective. Psychol. Bull. 141, 655–693. doi: 10.1037/a0038648 25621978

[ref81] Rentfrow P. J. Gosling S. D. (2003). The do re mi’s of everyday life: the structure and personality correlates of music. J. Pers. Soc. Psychol. 84, 1236–1256. doi: 10.1037/0022-3514.84.6.1236, 12793587

[ref82] Russell J. A. (1980). A circumplex model of affect. J. Pers. Soc. Psychol. 39, 1161–1178. doi: 10.1037/h0077714

[ref83] Ryff C. D. (2022). Positive psychology: looking back and looking forward. Front. Psychol. 13:840062. doi: 10.3389/fpsyg.2022.840062, 35369156 PMC8967995

[ref84] Saarikallio S. H. (2008). Music in mood regulation: initial scale development. Music. Sci. 12, 291–309. doi: 10.1177/102986490801200206

[ref85] Saarikallio S. Alluri V. Maksimainen J. Toiviainen P. (2021). Emotions of music listening in Finland and in India: comparison of an individualistic and a collectivistic culture. Psychol. Music 49, 989–1005. doi: 10.1177/0305735620917730

[ref86] Saarikallio S. Erkkilä J. (2007). The role of music in adolescents’ mood regulation. Psychol. Music 35, 88–109. doi: 10.1177/0305735607068889

[ref87] Saarikallio S. Randall W. M. Baltazar M. (2020). Music listening for supporting adolescents’ sense of agency in daily life. Front. Psychol. 10:2911. doi: 10.3389/fpsyg.2019.02911, 32010014 PMC6960221

[ref88] Saccaro L. F. Giff A. De Rossi M. M. Piguet C. (2024). Interventions targeting emotion regulation: a systematic umbrella review. J. Psychiatr. Res. 174, 263–274. doi: 10.1016/j.jpsychires.2024.04.025, 38677089

[ref89] Seligman M. E. P. Csikszentmihalyi M. (2000). Positive psychology: an introduction. Am. Psychol. 55, 5–14. doi: 10.1037/0003-066X.55.1.5 11392865

[ref90] Sheppes G. Meiran N. (2008). Divergent cognitive costs for online forms of reappraisal and distraction. Emotion 8, 870–874. doi: 10.1037/a0013711, 19102598

[ref91] Sheppes G. Suri G. Gross J. J. (2015). Emotion regulation and psychopathology. Annu. Rev. Clin. Psychol. 11, 379–405. doi: 10.1146/annurev-clinpsy-032814-112739, 25581242

[ref92] Speer M. E. Ibrahim S. Schiller D. Delgado M. R. (2021). Finding positive meaning in memories of negative events adaptively updates memory. Natl. Commun. Assoc. 12:6601. doi: 10.1038/s41467-021-26906-4, 34782605 PMC8593143

[ref93] Sweeny K. Dooley M. D. (2017). The surprising upsides of worry. Soc. Personal. Psychol. Compass 11, 1–10. doi: 10.1111/spc3.12311, 40046247

[ref94] Tabibnia G. (2020). An affective neuroscience model of boosting resilience in adults. Neurosci. Biobehav. Rev. 115, 321–350. doi: 10.1016/j.neubiorev.2020.05.005, 32522489

[ref95] Tang Q. Han J. Zeng X. (2024). The impacts of background music on the effects of loving-kindness meditation on positive emotions. Behav. Sci. (2076-328X) 14:204. doi: 10.3390/bs14030204, 38540508 PMC10968550

[ref96] Thayer R. (1989). The Biopsychology of mood and Arousal. Oxford: Oxford University Press.

[ref97] Thomas L. Kumar V. V. (2026). Headphones on, world off: exploring music as a tool for mood regulation, mental focus, and learning among teenagers. Safer Communities 25, 177–193. doi: 10.1108/SC-05-2025-0035

[ref98] Torlak M. S. Ünüvar B. S. Tüfekçi O. Gerçek H. Dursun B. (2025). The effects of music listening on pain, anxiety, and quality of life in patients with chronic low back pain. Clin. Exp. Health Sci. 15, 333–339. doi: 10.33808/clinexphealthsci.1552871

[ref99] Ubillos-Landa S. Puente-Martínez A. (2026). The influence of negative emotions in coping with intimate partner violence against women over time. Psychol. Violence 16, 64–78. doi: 10.1037/vio0000613

[ref100] Van Goethem A. Sloboda J. A. (2011). The functions of music for affect regulation. Music. Sci. 15, 208–228. doi: 10.1177/1029864911401174

[ref101] Venkatesan T. Demetriou A. M. Hempel A. Bowling D. L. (2026). A scoping review of music-based digital therapeutics for stress, anxiety, and depression. Front. Hum. Neurosci. 20:1602004. doi: 10.3389/fnhum.2026.1602004, 41907803 PMC13021903

[ref102] Vuoskoski J. K. Eerola T. (2012). Can sad music really make you sad? Indirect measures of affective states induced by music and autobiographical memories. Psychol. Aesthet. Creat. Arts 6, 204–213. doi: 10.1037/a0026937

[ref103] Wang Y. Zai F. Zhou X. (2025). The impact of emotion regulation strategies on teachers’ well-being and positive emotions: a meta-analysis. Behav. Sci. 15:342. doi: 10.3390/bs15030342, 40150237 PMC11939169

[ref104] Watson D. Clark L. A. Tellegen A. (1988). Development and validation of brief measures of positive and negative affect: the PANAS scales. J. Pers. Soc. Psychol. 54, 1063–1070. doi: 10.1037/0022-3514.54.6.1063, 3397865

[ref105] Waugh C. E. (2020). The roles of positive emotion in the regulation of emotional responses to negative events. Emotion 20, 54–58. doi: 10.1037/EMO0000625, 31961178

[ref106] Wei Q. He W. (2026). The application of AI-assisted music therapy tools in mental health interventions. Front. Psychol. 17:1463. doi: 10.3389/fpsyg.2026.1741463, 41658379 PMC12874394

[ref107] Weth K. Rabb M. H. Carbon C. C. (2015). Investigating emotional responses to self-selected sad music via self-report and automated facial analysis. Music. Sci. 19, 412–432. doi: 10.1177/1029864915606796

[ref108] Wilson K. G. DuFrene T. (2009). Mindfulness for Two: An Acceptance and Commitment Therapy Approach to Mindfulness in Psychotherapy. Oakland, CA: New Harbinger.

[ref109] Wimbarti S. Tallei T. E. Homenta Rampengan D. D. C. Nababan S. P. Rampengan J. A. C. H. Tjandra K. C. . (2025). Efficacy of music listening in alleviating anxiety among college students: a meta-analysis of randomized controlled trials. Curr. Psychol. 44, 16400–16411. doi: 10.1007/s12144-025-08353-2, 30311153

[ref110] Witten E. Ryynanen J. Wisdom S. Tipp C. Chan S. W. Y. (2023). Effects of soothing images and soothing sounds on mood and well-being. Br. J. Clin. Psychol. 62, 158–179. doi: 10.1111/bjc.12400, 36342851

[ref111] Wong P. T. P. (2019). Second wave positive psychology’s (PP 2.0) contribution to counselling psychology. Couns. Psychol. Q. 32, 275–284. doi: 10.1080/09515070.2019.1671320

[ref112] Xie D. Lu J. Xie Z. (2023). Emotion experience and regulation in undergraduates following social rejection: a daily diary study. Soc. Behav. Personal. Int. J. 51, 1–10. doi: 10.2224/sbp.12609

[ref113] Yamamoto M. Naga S. Shimizu J. (2007). Positive musical effects on two types of negative stressful interventions. Psychol. Music 35, 249–275. doi: 10.1177/0305735607070375

[ref114] Yaribeygi H. Panahi Y. Sahraei H. Johnston T. P. Sahebkar A. (2017). The impact of stress on body function: a review. EXCLI J. 16, 1057–1072. 10.17179/excli2017-480, 28900385 PMC5579396

[ref115] Yehuda N. (2011). Music and stress. J. Adult Dev. 18, 85–94. doi: 10.1007/s10804-010-9117-4

[ref116] Zander-Schellenberg T. Collins I. M. Miché M. Guttmann C. Lieb R. Wahl K. (2020). Does laughing have a stress-buffering effect in daily life? An intensive longitudinal study. PLoS One 15, 1–11. doi: 10.1371/journal.pone.0235851, 32645063 PMC7347187

[ref117] Zentner M. Grandjean D. Scherer K. R. (2008). Emotions evoked by the sound of music: characterization, classification, and measurement. Emotion 8, 494–521. doi: 10.1037/1528-3542.8.4.494, 18729581

[ref118] Zhang Q. Ge Y. Qu W. (2024). The effect of relaxing music on driving anger and performance in a simulated car-following task. Human Factors Ergon. Manuf. Serv. Ind. 34, 386–395. doi: 10.1002/hfm.21031

